# Maximizing citizen scientists’ contribution to automated species recognition

**DOI:** 10.1038/s41598-022-11257-x

**Published:** 2022-05-10

**Authors:** Wouter Koch, Laurens Hogeweg, Erlend B. Nilsen, Anders G. Finstad

**Affiliations:** 1 Department of Natural History, Norwegian University of Science and Technology, Trondheim, Norway; 2 Norwegian Biodiversity Information Centre, Trondheim, Norway; 3 Intel Benelux, High Tech Campus 83, 5656 AE Eindhoven, The Netherlands; 4 Naturalis Biodiversity Center, PO Box 9517, 2300 RA Leiden, The Netherlands; 5 Faculty of Biosciences and Aquaculture, Nord University, Steinkjer, Norway

**Keywords:** Biodiversity, Machine learning, Data acquisition

## Abstract

Technological advances and data availability have enabled artificial intelligence-driven tools that can increasingly successfully assist in identifying species from images. Especially within citizen science, an emerging source of information filling the knowledge gaps needed to solve the biodiversity crisis, such tools can allow participants to recognize and report more poorly known species. This can be an important tool in addressing the substantial taxonomic bias in biodiversity data, where broadly recognized, charismatic species are highly over-represented. Meanwhile, the recognition models are trained using the same biased data, so it is important to consider what additional images are needed to improve recognition models. In this study, we investigated how the amount of training data influenced the performance of species recognition models for various taxa. We utilized a large citizen science dataset collected in Norway, where images are added independently from identification. We demonstrate that while adding images of currently under-represented taxa will generally improve recognition models more, there are important deviations from this general pattern. Thus, a more focused prioritization of data collection beyond the basic paradigm that “more is better” is likely to significantly improve species recognition models and advance the representativeness of biodiversity data.

## Introduction

Addressing the current crisis related to biodiversity loss necessarily involves addressing several fundamental knowledge gaps^1,2^. Currently there are vast spatial, temporal and especially taxonomic gaps and biases in global primary biodiversity data sets, limiting our understanding of the earth’s biosphere^3–6^. Several observation methods based on image recognition, ranging from remotely operated vessels to camera traps and citizen science programs^7–9^, hold great promise in solving some of the taxonomic biases currently experienced^10^. Citizen science (observations made by non-professional volunteers^11^) has emerged as a very large source of biodiversity data. It has the potential to fill gaps in our current knowledge about the occurrence of species in time and space^12–14^. Several citizen science programs, e.g. iNaturalist, eBird, iSpot^15^ contribute data on vast scales and in amounts that cannot feasibly be acquired in any other way. Such programs come with the added benefit of educating and engaging the general public^16–18^. Some of the main concerns related to citizen science data are reliability of the taxon identifications reported^19,20^, and the over-representation of more charismatic taxa such as birds and flowering plants^21–23^. Improving the quality of citizen science data is a vital step in addressing the knowledge gaps in our understanding of the earth’s biosphere.

Image recognition models can help citizen scientists recognize more species and provide a quality control mechanism that helps to reduce the risk of species misidentification^10^. Their performance is however inherently linked to the quality of the data used to train them. By increasingly helping citizen scientists identify species from images^24–26^, such tools help address the aforementioned issues in citizen science data, adding to the quality, quantity and taxonomic scope of observations. Image recognition models can warn the citizen scientist and validators of potential misclassifications. Output of citizen scientists is increased as automating parts of the reporting process makes reporting less time consuming. Image recognition models also allow citizen scientists to report more of what they encounter by enabling them to report taxa they could not have identified independently. The taxonomic scope of the citizen scientist is expanded when tools enable them to identify and report within taxa they would otherwise not be familiar with^27^. Observations accompanied by images can be used for training an image recognition model for use in the field. Generally one aims to keep training data as similar as possible to the intended classification task of the model^28^. By using images from citizen scientists when training a model intended for use within citizen science, one is more likely to capture the variability in the kind of images provided by citizen scientists. Images from other sources may be more standardized, depict close-ups of relevant features, and/or depict preserved and prepared specimens. Deep neural networks are designed to draw inferences from novel data by generalized patterns observed in training data^28^, requiring substantial amounts of data. The Computer Vision model by iNaturalist, for example, only includes taxa for which at least 100 images are available^29^. This criterion excludes 89% of the taxa with at least one image in the dataset used in this study, illustrating how heavily the training of models depends on the amounts of data citizen science provides. In this manner, citizen science and automated image recognition are increasingly interdependent. Image recognition models help citizen scientists collect data to expand our knowledge base, whilst training of the next generation of recognition models depends on the collection of more images.

While some species are readily recognized with limited experience, others require extensive experience with many specimens to obtain the necessary knowledge. The distinct colorations of butterflies may allow any interested observer with some experience to reliably identify the majority of species in certain areas, while a taxon like Diptera remains notoriously difficult even after years of study. Machine learning is no different from human learning in this respect; different amounts of training data are required depending on the distinctness of species’ characteristics. Therefore, there can be substantial differences between taxa in the number of images required per species for the best achievable model performance. This can depend on factors like species’ distinctiveness, the variation in appearance, the various angles and contexts in which photos are taken, and the extent to which a species’ behavior is suited for high quality documentation^27,30,31^. As a result, the value of adding a new image to the training set is not equal across taxa, but varies both because the size of the existing training set is different, and the fact that some species are more distinct than others. Thus it is important to consider the informational value of adding images to the training data.

In this study, we use the Species Observation Service, a large Norwegian citizen science project, as an example to investigate the nature of the bias in citizen science image data, and how this relates to the value of data for image recognition models. One way to evaluate this is by using the concept of Value of Information (VoI); “*the increase in expected value that arises from making the best choice with the benefit of a piece of information compared to the best choice without the benefit of that same information*”^32^. Considering training data for image recognition models in the VoI framework allows us to identify the most effective prioritization for improving recognition models. This method allows for a more sophisticated approach to data collection than simply adding more data for all taxa, or even for taxa that are currently the most under-represented. First, we evaluate whether the biases generally found in all observation data, regardless of source, are the same within citizen science observations with images, or if there are different biases that need to be taken into account. Then we train multiple image recognition models for different taxa, with a gradually increasing number of images per species, allowing us to quantify and compare the effects of adding more training data between taxa. Using these changes in performance, we estimate the VoI of adding training data for each taxon, relative to the amount of images that are currently available. Finally, comparing this VoI to the amount of over- or under-representation of these taxa, we demonstrate that mobilizing images with a higher VoI provides an alternative, data-driven and efficient approach compared to simply prioritizing images of the currently most under-represented taxa.

## Results

### Taxonomic bias in citizen science observations with or without images

**Figure 1:**
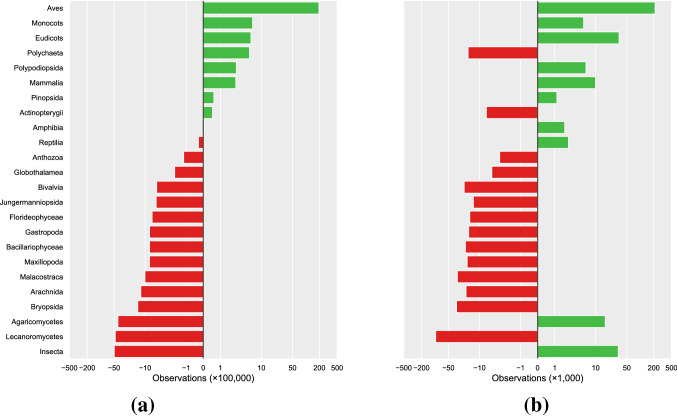
The per-species representation of observations in Norway per class, using all GBIF data (**a**) or only GBIF mediated citizen science data with images (**b**). The 0-line is where the values would be if the average number of observations per species in that class was equal to the average number of observations per species over all classes combined. Plotted here on an inverse hyperbolic sine-transformed scale, sorted by the per-species representation in subplot (**a**).

It has been well documented in a global context that particularly charismatic taxonomic classes have many times more reported observations per species than those that are considered less charismatic^5^. We find the same pattern when considering classes within the totality of GBIF mediated observations for Norway from all sources (Fig. 1a). When limiting this analysis to only observations with images that originate from the citizen science platform Species Observation Service^33^, a different pattern emerges (Fig. 1b). Perhaps most eye-catchingly, Insecta are the most under-represented taxon in the totality of Norwegian observations, but the 3rd most over-represented when limiting the analysis to citizen science images. We performed a similar analysis for the 12 taxonomic orders used in the machine learning part of this study. This provides the biases in relative representation per species in the data available for training our recognition models.

### Image recognition performance and the value of information

**Figure 2:**
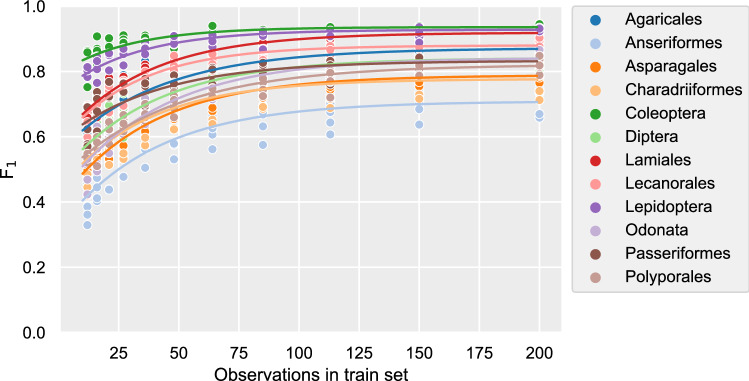
The performance (F$_1$ score) vs the training set size. Lines are the fitted Von Bertalanffy Growth Function-curves per order. See the Supplementary Information for an interpretation of such curves.

**Figure 3:**
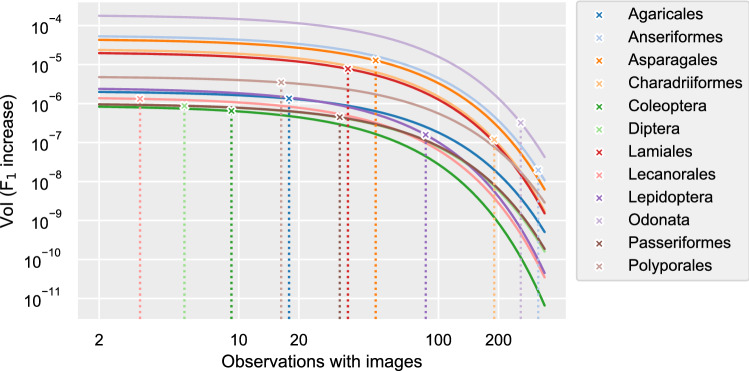
The VoI (F$_1$ increase) for each order as the result of adding a single observation with at least one image for a single species, versus the average number of observations with images available per species. Dotted lines mark the average number of observations with images per species currently available for the respective order, from which the current expected VoI (marked with ✕) is derived.

When training image recognition models, the amount of training data provided to the model determines how well the model is able to recognize species in the test images. For all orders, as models are provided with more images per species, their performance (as measured by the F$_1$ scores) increases. Comparing the performances for each order at the lowest and highest number of training images per species, as well as the gradual performance increase over intermediate numbers of training images, it is clear that the 12 orders have distinct performance curves (Fig. 2). From this it follows that the increase in performance at any given point on these curves—the value of information (VoI) of adding observations with images at that point—also differs between orders. Combined with substantially different amounts of currently available observations between orders, the estimated VoI of adding an observation with at least one image to those currently available for that order also varies widely (Fig. 3).

### Combining value of information and taxonomic bias

**Figure 4:**
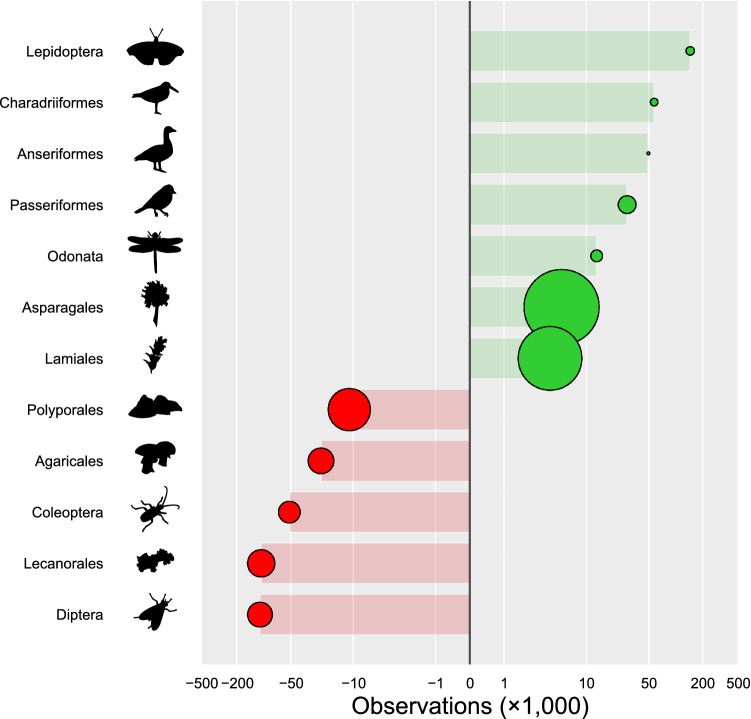
The relative per-species representation in Norwegian citizen science observations with images, and their value of information. The areas of the circles are relative to their respective VoI, defined as the current expected performance increase (in F$_1$ score) for one added observation with images for that order. If the VoI of adding data was mainly determined by the current relative over- or under-representation of a taxon, one would expect circles to gradually increase for more under-represented orders in the lower part of the graph. Numerical values provided in the Supplementary Information.

After obtaining the per-species over- and under-representations, as well as the current expected VoI of additional observations with images, we can compare the two values for each order in the experiment. Plotting the taxonomic bias of the orders used in this experiment together with their estimates for their respective estimated VoI, it is clear that current under- or over-representation of the order is not the determining factor for the expected value of additional observations. While the VoI of under-represented orders is generally higher, differences between orders in their learning curves cause some orders to have a higher or lower VoI than just their overall over- or under-representation would indicate (Fig. 4).

## Discussion

We set out to investigate the taxonomic bias in citizen science data, in particular when accompanied by images, using a large Norwegian citizen science project as an example case. Such images can be used to train deep neural networks for image recognition, helping citizen scientists by verifying species identifications and addressing some of the inherent taxonomic bias as they then can report within taxa they are not able to identify independently. By examining how the performance of recognition models increases as they are provided with more images in an experimental setup, we can estimate how much we expect models to improve when adding more images to those currently available for each taxon. Comparing this value of information (VoI) to the taxonomic bias within citizen science image data, we propose data prioritization strategies based on what additional data would improve recognition models the most. Such strategies would be more efficient than merely focusing more on taxa for which there are currently fewer images available.

### Taxonomic bias

The taxonomic biases within citizen science observations considered in the current study follow a similar pattern to what has been found across biodiversity data in general^5^. However, when only considering citizen science observations with images, these trends are less pronounced; plants and fungi have relatively higher percentages of observations with images than for example birds (Fig. 1b). This indicates that while birds are still the most reported group also within citizen science observations with images, bird observations are generally less commonly documented with images. The reverse is true for the Insecta, which are so abundant in the citizen science image data as to be the 3rd most overrepresented class in that context. This is in stark contrast to what has been found for the totality of GBIF mediated observations globally^5^ and in the Norwegian context we examined here, where the Insecta are the single most under-represented class.

Analyzing the taxonomic biases for the orders used in the machine learning part of this study sheds some light on the underlying mechanisms. While all orders within Aves are over-represented regardless of the nature of the observations considered, the Insecta are more diverse in their bias, as illustrated by Lepidoptera being the most over-represented order but Diptera the most under-represented.

We hypothesize that this disparity between taxonomic bias in all data versus that in citizen science data with images is most likely a combination of the behavior of the species and the kind of citizen scientists reporting the observations. There are distinctly different types of citizen scientists, with their own contribution patterns^34^. For casual reporters lacking specialized equipment, charismatic butterflies and flowering plants are more readily photographed opportunistically than birds. Meanwhile, a group of quite persistent ornithologists report the bulk of the bird observations in the dataset. This is typically a group reporting in a structured manner, more often based on local inventories and checklists, where reporting with images is less common than with opportunistic observations.

### Image recognition and value of information

There are clearly differences between orders in the rates at which image recognition improves as more images are made available per species (Fig. 2). These differences between orders manifest in both initial performances, the rate at which performances change, and the maximum performance achieved. This indicates that, as is the case for humans, it requires more experience to learn to identify species within certain taxa than others, while the reliability with which species are correctly identified once the necessary knowledge has been acquired also differs. The differences between orders in this regard is not necessarily directly linked to the taxon’s characteristics alone, however. Image quality and composition can vary between taxa depending on factors such as specimens’ behavior or lack thereof, physical size, and the kind of citizen scientist generally photographing the species. A stationary flower is easier to photograph with a lot of detail than a centipede running for cover. A mite that can only be photographed with a macro lens will be photographed by a citizen scientist who has invested in such equipment. This type of citizen scientist is also more likely to invest time in taking a high quality picture than a casual citizen scientist snapping a squirrel with their mobile phone.

The VoI estimates for each of the orders provides equally diverse results. For any given number of images per species, orders differ in the expected performance increase at that point, as do the relative rates at which these performances change as data is added. As a consequence, there is a range of varying estimates for the VoI for each order, depending on both the number of images currently available per species, and the way the VoI per additional observation with images declines as more images are already available to the model.

### Combining taxonomic biases and the value of information

We now have an estimate of how over- or under-represented the orders with which the recognition models have been trained are relative to one another, as well as a per-order estimate of the VoI per added observation with images. This means that we can address the question whether models are best improved by adding more image data equally across orders, if one should ideally prioritize under-represented orders, or if there is a prioritization to be made based on order-specific differences. As shown in Fig. 4, there are distinct differences in the VoI per order, and these do not merely correlate with their respective over- or under-representation. The plant orders of Asparagales and Lamiales clearly have a higher VoI despite their slight over-representation when compared to the other orders in this experiment. The fungi order Polyporales also gains more than twice the VoI per additional observation with images in comparison to the fourth-most valuable order, the Lecanorales. We conclude that, from a VoI perspective, these are the orders for which a recognition model would benefit the most per observation with images added, despite the fact that other orders are numerically more under-represented.

## Conclusions

Based on the value of information (VoI) for image recognition models, a citizen scientist or citizen science project manager aiming to maximize their impact in this regard might want to focus on orders with the highest expected VoI per observation with images added, rather than simply on the order with the lowest number of images per species. Observations with images of other orders, while in some cases less well-represented in the available image data, appear to provide less VoI per additional observation. As citizen scientists are in large part motivated by a desire to advance scientific knowledge^35^, communicating such considerations can be an important part of community engagement.

In generalizing these findings, the following has to be noted:

- The taxa identified here as having the highest expected VoI per observation with images added are examples from the limited subset of orders used within this experiment. As illustrated by the observed variation in per-species representation and VoI between orders that belong to the same class, it is evident that generalization of a class like Insecta fails to give insight into intra-class variation. It is likely that a similar principle applies to orders, where for example a taxonomic group like Norwegian warblers likely has a different VoI curve than the more readily distinguished titmice. Such differences will remain hidden from view when analyzing passerine birds as a single taxonomic group.
- Our findings are derived from Norwegian species reported on a single Norwegian citizen science portal. The diversity of species within the same orders can differ in other regions, affecting the VoI curves. Different portals will also differ in the way they accommodate reporting observations with images, and in general attract different types of users^23^. All of these factors are likely to have an effect on the proportion of observations accompanied by photographic evidence and the quality thereof. Such factors also affect the nature of newly added data, including its expected VoI.
- Models were trained on species for which at least 220 observations with images were available. This is not a random subset of all the species within an order, and likely to be biased towards charismatic species and those that are more readily identifiable from an image. This can lead to an overestimation in terms of learning rate and thus the VoI curve, especially within orders in which relatively few species have the data availability we selected for here. Then again, future observations to be added to the data will be prone to the same biases, in which case the VoI of such an addition will be lower than it would be for a truly random species.
- Current and future (deep) learning methods alternative to Convolutional Neural Networks (CNN) may be able to utilize more information in an image and generalize more rapidly, using less data. This could have implications for the importance of VoI relative to the overall bias. We expect that the demonstrated differences in VoI between species are not unique to CNN however, and in part inherent to the visual information available in each picture. Either way, awareness of the potential differences in VoI between taxa is warranted, and an interesting consideration to evaluate in future studies.

Regardless of the specific taxa and derived values, our findings demonstrate that a more informed decision is possible when choosing to focus on certain taxa for data collection aimed at improved recognition models. Prioritization of taxa for which to mobilize additional data can be informed by considering its expected VoI, rather than simply prioritizing those that are currently the most under-represented numerically. Note that this is no plea for deprioritizing data collection for such taxa in the context of citizen science as a whole. There are many areas of management and research that can benefit from additional data on taxa we predict will benefit less from additional images for recognition models, and ample reasons to mobilize data for other applications than image recognition.

Training machine learning models requires substantial amounts of data, certainly when context, morphology and phenology vary, such as when classifying in situ images. Data collection in machine learning generally is a matter of harvesting whatever one can to provide the model with more data. Within (citizen) science, the collection of images mainly serves as secondary data, providing documentation for the occurrence it accompanies. With the more widespread use of image recognition models as both a user tool and a mechanism for quality control, it is time to view images as data in and of themselves. Such a shift calls not only for conscious choices when it comes to the VoI in images, but increased implementation of data practices such as persistent storage, metadata standardization and the other FAIR data principles^36^ to enable more apt usage of image data for current and novel applications.

## Methods

In the current study we utilize an extensive network and data from citizen science in order to test for among taxa variation in biases and value of information (VoI) in image recognition training data. We use data from the Norwegian Species Observation Service as an example dataset due to the generic nature of this citizen science platform, where all multicellular taxa from any Norwegian region can be reported both with and without images. The platform is open to anyone willing to report under their full real name, and does not record users’ expertise or profession. The platform had 6,205 active contributors in 2021 out of its 17,655 registered users, and currently publishes almost 27 million observations through GBIF, of which 1.08 million with one or more images. Observations have been bulk-verified by experts appointed by biological societies receiving funding for this task, with particular focus on red listed species, invasive alien species, and observations out of range or season. Observations containing pictures receive additional scrutiny, as other users can alert reporters and validators to possible mistaken identifications. An advantage of this particular platform is that no image recognition model has been integrated. This ensures that the models trained in this experiment are not trained on the output resulting from the use of any model, but with identifications and taxonomic biases springing from the knowledge and interest of human observers. Moreover, the platform’s compliance with the authoritative Norwegian taxonomy allows for analyses on taxonomic coverage.

In an exploration procedure we determined the taxonomic level of orders to be suitable examples of taxa with a sufficiently wide taxonomic diversity, and enough data in the dataset to be evaluated for models in this experiment. Data collection was done by acquiring taxon statistics and observation data from the Global Biodiversity Information Facility (GBIF), the largest aggregator of biodiversity observations in the world^37^ for the selected orders, as well as the classes used by Troudet et al.^5^. The authoritative taxonomy for Norway was downloaded from the Norwegian Biodiversity Information Centre^38^. In the experimental procedure, models were trained for 12 distinct orders (listed in Fig. 4), artificially restricting these models to different amounts of data. In the data analysis stage, model performances relative to the amount of training data were fitted for each order, allowing the estimation of a VoI. Using the number of observations per species on GBIF, and the number of species known to be present in Norway from the Norwegian Species Nomenclature Database, we calculated relative taxonomic biases.

### Exploration

Initial pilot runs were done on 8 taxa (see Supplementary Information), using different subset sizes of observations for each species, and training using both an Inception-ResNet-v2^39^ as well as an EfficientNetB3^40^ architecture for each of these subsets. These initial results indicated that the Inception-ResNet-v2 performance (F$_1$) varied less between replicate runs and was generally higher, so subsequent experiments were done using this architecture. The number of observations which still improved the accuracy of the model was found to be between 150 and 200 in the most extreme cases, so the availability of at least 220 observations with images per species was chosen as an inclusion criteria for the further experiment. This enabled us to set aside at least 20 observations per species as a test dataset for independent model analysis.

From a Darwin Core Archive file of Norwegian citizen science observations from the Species Observation Service with at least one image^33^, a tally of the number of such observations per species was generated. We then calculated how many species, with a minimum of 220 such observations, would, at a minimum, be available per taxon if a grouping was made based on each taxon rank level with the constraint of resulting in at least 12 distinct taxa. For each taxonomic level, we calculated how many species having at least 220 such observations were available per taxon when dividing species based on that taxon level. When deciding on the appropriate taxon level to use, we limited the options to taxon levels resulting in at least 12 different taxa.

A division by order was found to provide the highest minimum number of species (17) per order within these constraints, covering 12 of the 96 eligible orders. The next best alternative was the family level, which would contain 15 species per family, covering 12 of the 267 eligible families.

### Data collection

We retrieved the number of species represented in the Norwegian data through the GBIF API, for all observations, all citizen science observations, and all citizen science observations with images for the 12 selected orders and the classes used by Troudet et al.^5^. We also downloaded the Norwegian Species Nomenclature Database^38^ for all kingdoms containing taxa included in these datasets. Observations with images were collected from the Darwin Core Archive file used in the exploration phase, filtering on the selected orders. For these orders, all images were downloaded and stored locally. The average number of images per observation in this dataset was 1.44, with a maximum of 17 and a median of 1.

### Experimental procedure

**Figure 5:**
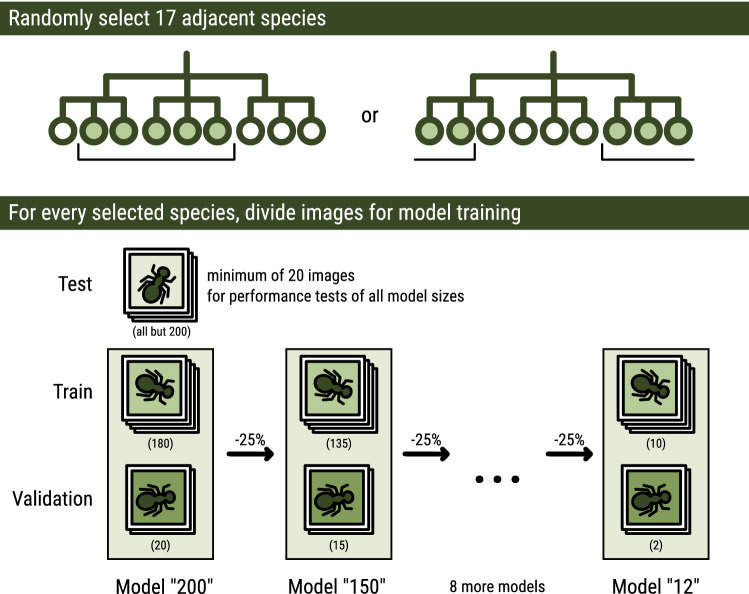
Data selection and subdivision. Each run is generated by selecting 17 taxonomically adjacent species per order, and randomly assigning all available images of each selected species to that run’s test-, train- or validation set. Training data are used as input during training, using the validation data to evaluate performance after each training round in order to adjust training parameters during training. The test set is used to measure model performance independently after the model is finalized^28^. For each subsequent model in that run, training and validation data are reduced by 25% (or slightly less than 25% if not divisible by 4). The test set is not reduced, and used for all models within a run.

For each selected order, a list of all species with at least 220 observations with images was generated from the Darwin Core Archive file^33^. Then, runs were generated according to the following protocol (Fig. 5):

1. From a list sorted alphabetically by the full taxonomy of the species, a subset of 17 consecutive species starting from a random index was selected. If the end of the list was reached with fewer than 17 species selected, selection continued from the start of the list. The taxonomic sorting ensures that closely related species (belonging to the same family or genus), bearing more similarity, are more likely to be part of the same experimental set. This ensures that the classification task is not simplified for taxa with many eligible species.
2. Each of the 220+ observations for each species were tagged as being either test, training or validation data. A random subset of all but 200 were assigned to the test set. The remaining 200 observations were, in a 9:1 ratio, randomly designated as training or validation data, respectively. In all cases, images from the same observation were assigned to the same subset, to keep the information in each subset independent from the others. The resulting lists of images are stored as the test set and 200-observation task.
3. The 200 observations in the training and validation sets were then repeatedly reduced by discarding a random subset of 25% of both, maintaining a validation data proportion of $\le$10%. The resulting set was saved as the next task, and this step was repeated as long as the resulting task contained a minimum of 10 observations per species. The test set remained unaltered throughout.

Following this protocol results in a single run of related training tasks with 200, 150, 113, 85, 64, 48, 36, 27, 21, 16 and 12 observations for training and validation per species. The seeds for the randomization for both the selection of the species and for the subsetting of training- and validation datasets were stored for reproducibility. The generation of runs was repeated 5 times per order to generate runs containing tasks with different species subsets and different observation subsetting.

Then, a Convolutional Neural Network based on Inception-ResNet-v2^39^ (see the Supplementary Information for model configuration) was trained using each predesignated training/validation split. When the learning rate had reached its minimum and accuracy no longer improved on the validation data, training was stopped and the best performing model was saved. Following this protocol, each of the 12 orders were trained in 5 separate runs containing 11 training tasks each, thus producing a total of 660 recognition models. After training, each model was tested on all available test images for the relevant run.

### Data analysis

The relative representation of species within different taxa were generated using the number of species present in the GBIF data for Norway within each taxon and the number of accepted species within that taxon present in the Norwegian Species Nomenclature Database^38^, in line with Troudet et al.^5^: $R_x = n_x - (n \frac{s_x}{s})$ where $R_x$ is the relative representation for taxon $x$, $n_x$ is the number of observations for taxon $x$, $n$ is the total number of observations for all taxa, $s_x$ is the number of species within taxon $x$, and $s$ is the total number of species within all taxa.

As a measure of model performance, we use the F$_1$ score, the harmonic mean of the model’s precision and recall, given by

$$\begin{aligned} F_1 = \frac{tp}{tp + \frac{1}{2}(fp + fn)} \end{aligned}$$

where $tp$, $fp$ and $fn$ stand for true positives, false positives and false negatives, respectively. The F$_1$ score is a commonly used metric for model evaluation, as it is less susceptible to data imbalance than model accuracy^28^.

The value of information (VoI) can be generically defined as “*the increase in expected value that arises from making the best choice with the benefit of a piece of information compared to the best choice without the benefit of that same information*”^32^. In the current context, we define the VoI as the expected increase in model performance (F$_1$ score) when adding one observation with at least one image. To estimate this, for every order included in the experiment, the increase in average F$_1$ score over increasing training task sizes were fitted using the Von Bertalanffy Growth Function, given by

$$\begin{aligned} L = L_\infty (1 - e^{-k(t-t_0)}) \end{aligned}$$

where $L$ is the average F$_1$ score, $L_\infty$ is the asymptotic maximum F$_1$ score, $k$ is the growth rate, $t$ is the number of observations per species, and $t_0$ is a hypothetical number of observations at which the F$_1$ score is 0. The Von Bertalanffy curve was chosen as it contains a limited number of parameters which are intuitive to interpret, and fits the growth of model performance well.

The estimated increase in performance at any given point is then given by the slope of this function, i.e. the result of the differentiation of the Von Bertalanffy Growth Curve, given^41^ by

$$\begin{aligned} \frac{dL}{dt} = bke^{-kt} \end{aligned}$$

where

$$\begin{aligned} b = L_\infty e^{kt_0} \end{aligned}$$

Using this derivative function, we can estimate the expected performance increase stemming from one additional observation with images for each of the species within the order. Filling in the average number of citizen science observations with images per Norwegian species in that order for t, and dividing the result by the total number of Norwegian species within the order, provides the VoI of one additional observation with images for that order, expressed as an average expected F$_1$ increase.

## Supplementary Information

Supplementary Information.

## Data Availability

The datasets generated and/or analyzed during the current study are available in the GBIF repository, https://doi.org/10.15468/dl.tc4w55.

## Code availability

All code used in this study for the experiment and the generation of this manuscript and its graphs is available on https://github.com/WouterKoch/citizen_science_VoI.
