# Supplementary material for: Maximizing citizen scientists’ contribution to automated species recognition
Source: Sci Rep. 2022 May 10;12:7648. doi: 10.1038/s41598-022-11257-x (PMC9090737; doi:10.1038/s41598-022-11257-x)
Supplement: Supplementary file 1 — Supplementary Information. [file 41598_2022_11257_MOESM1_ESM.pdf]

Supplementary Information

Pilot run taxa

Initial pilot runs were done on Bombus, Cetoniidae, Coccinellidae, Coleoptera, Lepidoptera, Odonata, Rodentia, and Zygaenidae. These taxa were chosen in order to test the machine learning pipeline on taxa with different levels of difficulty of identification. Rodentia were added to include a taxon outside of the Insecta.

Image recognition model configuration

Models were trained in Python 3.9[1], using TensorFlow[2] and Keras[3] to train a new recognition model based on the Inception-ResNet-v2 architecture[4] for every dataset. A dense classification layer using softmax activation replaced the top layer of the Inception-ResNet-v2 model as a new top layer, with 17 nodes to classify each of the 17 species. For the loss function we used standard categorical cross entropy loss.

Color channels of input images were normalized between -1 and 1, and were scaled to 256×256 pixels, cropping the image to become square if needed. Training data were augmented by shearing up to a factor of 0.2, zooming up to a factor of 0.2, rotating up to 90 degrees, and randomly flipping horizontally or not. Validation and test images were only normalized and squared, not augmented.

In the first training stage, the weights of the original Inception-ResNet-v2 layers were frozen, training only the newly added top layer. This was done for 2 epochs with a learning rate of $1 \cdot 10^{-3}$. This has an equivalent effect as learning rate warm-up.

In the second training stage, all layers were trained. This was done for a maximum of 200 epochs, with an initial learning rate of $1 \cdot 10^{-4}$. The learning rate was multiplied by 0.1 when the validation loss did not improve for 3 consecutive epochs. The minimum of the learning rate was set to $1 \cdot 10^{-8}$.

After each epoch, model performance was evaluated using the validation set, saving the weights of the current model to disk as the latest checkpoint if the accuracy for the validation set had improved since the last saved checkpoint. Finally, when the model did not reduce its loss for 8 consecutive epochs, training was stopped. The most recently stored checkpoint was then used as the final recognition model for that dataset, and its performance measured using the test data.

Taxonomic order result metrics

**Order**	**Bias in cs data with img**	**VoI (F_1 increase $\cdot 10^6$)**
Asparagales	5259	13.05
Lamiales	3879	9.36
Polyporales	-11060	4.11
Lecanorales	-106853	1.72
Agaricales	-22932	1.52
Diptera	-110248	1.42
Coleoptera	-51782	1.09
Passeriformes	28630	0.73
Odonata	13075	0.32
Lepidoptera	145110	0.17
Charadriiformes	57421	0.13
Anseriformes	49501	0.02

Table S1: Orders used in the machine learning experiment, their over- or under-representation among citizen science observations with images (relative to all orders having an equal average amount of such observations per species), and the Value of Information as measured by the expected F_1 increase for adding one observation with images to the number of observations with images currently available. Sorted by VoI (descending). These are the numerical values for figure **??**.

Von Bertalanffy Growth Curves

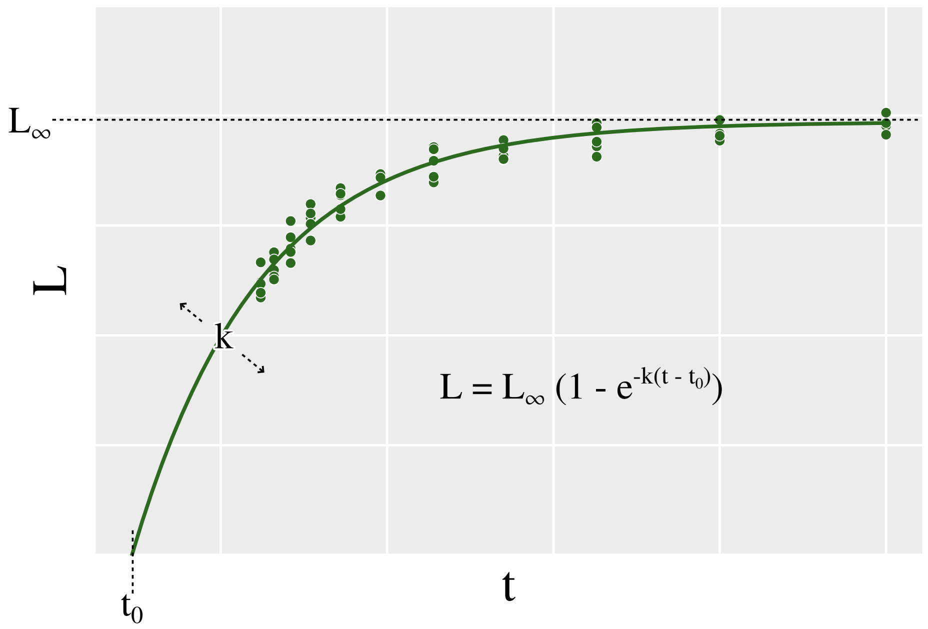

Figure S1: Visualization of the Von Bertalanffy Growth Curve parameters. Curves were fitted using the Levenberg-Marquardt (Least Squares) algorithm. Residuals were plotted for each taxon and not found to be heterogeneous in their distribution.

References

1. Python Software Foundation. *Python Language Reference, version 3.9* `http://www.python.org`.
2. Martín Abadi *et al. TensorFlow: Large-Scale Machine Learning on Heterogeneous Systems* Software available from tensorflow.org. 2015. `https://www.tensorflow.org/`.
3. Chollet, F. *et al. Keras* 2015. `https://keras.io`.
4. Szegedy, C., Ioffe, S., Vanhoucke, V. & Alemi, A. *Inception-v4, Inception-ResNet and the Impact of Residual Connections on Learning* 2016. arXiv: `1602.07261 [cs.CV]`.
